# A Novel *PCNT* Frame Shift Variant (c.7511delA) Causing Osteodysplastic Primordial Dwarfism of Majewski Type 2 (MOPD II)

**DOI:** 10.3389/fped.2020.00340

**Published:** 2020-06-25

**Authors:** Masoud Dehghan Tezerjani, Mohammad Yahya Vahidi Mehrjardi, Hossein Hozhabri, Masoud Rahmanian

**Affiliations:** ^1^Abortion Research Centre, Yazd Reproductive Sciences Institute, Shahid Sadoughi University of Medical Science, Yazd, Iran; ^2^Diabetes Research Center, Shahid Sadoughi University of Medical Sciences, Yazd, Iran; ^3^Department of Genetics, Shahid Sadoughi University of Medical Sciences, Yazd, Iran; ^4^Department of Experimental Medicine, Sapienza University of Rome, Rome, Italy

**Keywords:** MOPD II, birth defects, primordial dwarfism, *PCNT* gene, high-throughput nucleotide sequencing

## Abstract

**Background:** Microcephalic osteodysplastic primordial dwarfism type II (MOPD II) is an autosomal recessive and skeletal disorder included wide spectrum of clinical abnormalities such as fetal growth restriction, disproportionate face, microcephaly, post-natal growth retardation, adult height under 100 cm, abnormal skin pigmentation, insulin resistance, and susceptibility to cerebrovascular and hematologic abnormalities. Due to heterogeneous feature of MOPDs diseases and common clinical features among the different subtypes, mutation analysis can be considered as fundamental in the accurate diagnosis and confirmation of the MOPD II disease. Some studies revealed that, variants of gene encoding Pericentrin protein, *PCNT*, were associated with MOPD II.

**Methods:** We performed whole exome sequencing based on the next generation sequencing (Illumina platform), to perform correct diagnosis in a 17-year-old girl with an unknown disease who was referred to the Diabetes Research Center in Yazd, Iran. The clinical features of the patient were short stature, generalized brachydactyly, gradual deterioration of brain functioning, menstrual irregularity, clitoromegaly, acanthosis nigricans, diabetes mellitus, hyperinsulinemia, insulin resistance, and dyslipidemia. Accordingly, her parents were also first cousin with no background disease. After identifying the novel variant, it was confirmed in the proband and her family using bi-directional Sanger sequencing, and its pathogenicity was also checked by different online tools.

**Results:** Our study revealed a novel frame-shift variant in *PCNT* gene (c.7511delA, p.K2504Sfs^*^27), which causes premature termination of Pericentrin protein. The result disclosed that, the proband was affected by MOPD II disease. In addition, the Sanger sequencing confirmed the novel homozygote variant in the proband and heterozygote one in her parents, and the extended family perfectly segregated among them. Online tools such as Varsome and MutationTaster also showed a high level of pathogenicity for the variant identified.

**Conclusion:** A novel variant was identified in the proband and her extended family, which emphasized the importance of *PCNT* gene mutations analysis in the screening and accurate identification of MOPD II disease, especially in prenatal diagnosis.

## Introduction

Primordial dwarfism (PD), as a rare type of dwarfism, is known as a heterogeneous class of disorder associated with prenatal and postnatal growth retardation. Accordingly, it has been classified into 5 subtypes disorders as follows: Russell Silver syndrome, Meier-Gorlin syndrome, Seckel syndrome, Majewski Osteodysplastic Primordial Dwarfism (MOPD) type I/III, and MOPD II (1). Although most of the clinical features are common among these subtypes, only the individuals with Russell Silver syndrome have normal head size. In addition, the patients with Russell Silver syndrome or Meier–Gorlin syndrome are usually higher than other types of PD (2–4). MOPD, described by Majewski et al., have similar clinical conditions to Seckel syndrome such as prenatal and postnatal growth abnormalities and microcephaly. However, Majewski et al. discriminated between MOPD types and Seckel syndrome in terms of the severity; absence or presence of clinical conditions such as mental and growth retardation; and bone abnormalities (5). Majewski et al. also categorized MOPD to into three distinct types (MOPD I, II, III), whereas type I and III are now known as a same disorder. MOPD Type II (OMIM: 210720) is the most common type, inherited in an autosomal recessive mode (6, 7). The clinical abnormalities associated with MOPD II include fetal growth restriction, microcephaly, post-natal growth retardation, skeletal dysplasia, and disproportionate face (7, 8). In addition, their adult height is under 100 cm, and the average head circumference at the post-pubertal stage is 40 cm. Moreover, insulin resistance, truncal obesity, abnormal skin pigmentation, and hyperopia are other clinical symptoms in the individuals with MOPD type II (9, 10). The most important cause of death among these patients is ascribed to central nervous system (CNS) vascular anomalies, including moyamoya disease and aneurysms. Although those individuals suffering from MOPD II have smaller size of brain compared to healthy ones, most of them have intellectual ability near normal, unexpectedly (11). Furthermore, hematologic abnormalities such as anemia, leukocytosis, and thrombocytosis are frequent among the patients with the disease (12).

Numerous studies reported that, mutation variants in the *PCNT* gene created susceptibility to MOPD II (13, 14). Also, this gene encodes Pericentrin protein (~370 kD), which is an anchoring protein and localizes to the centrosome. In addition, it is a fundamental player in the regulation of cell cycle and mitotic spindle (15). Although the main molecular mechanisms for most of the clinical features of the MOPD II are still unclear, our knowledge on the genes, variations, and proteins associated with this disease is increasing using high-throughput sequencing technology.

In this study, we reported a novel homozygous variant (c.7511delA) in exon 35 of the *PCNT* gene in an Iranian patient diagnosed with dyslipidemia and severe insulin resistance, which was finally diagnosed as MOPD II.

## Case Presentation

**Figure 1:**
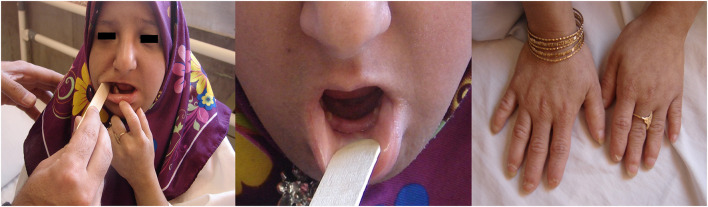
Left picture shows the frontal view of the patient showing microcephaly, prominent nose (wide bridge, broad root, columella under ala nasi), Middle picture shows Oligodontia, Right picture shows the Generalized brachydactyly **(**A written consent was obtained from the patient's parents to publish this image).

A 17-year-old girl (case IV-3) was referred to Yazd Diabetes Research Centre due to polyuria and polydipsia. In order to manage hyperglycemia, the patient's clinical features and family history were thoroughly examined by a physician and a genetic counselor. Her parents were first cousins, and she was the result of first pregnancy by vaginal delivery at 36 weeks gestation. Her mother have had normal pregnancy with no history of abortion or the related complications such as fetal decelerations and bleeding. Her weight, length, and head circumference at birth were 2100 g (<20th percentile), 40 cm (-3SD), and 29 cm (-2SD), respectively. She grew up near normal cognitive function; however, she was not able to learn at school. In this regard, she had also growth problem during childhood, and the results of assessments of infantile and childhood charts showed that, she was on the lower level than the normal percentile. Fasting Plasma Glucose (FPG) was indicated to be 340 by laboratory evaluation. The investigation also revealed a negative result for the history of Type 1 and Type 2 diabetes mellitus in her family. In addition, clinical and paraclinical examinations revealed several abnormalities including microcephaly, beaked prominent nose with broad nasal bridge, dental abnormality(Oligodontia), short stature, generalized brachydactyly, gradual deterioration of brain functioning, menstrual irregularity, clitoromegaly, acanthosis nigricans, diabetes mellitus (DM), hyperinsulinemia, hypertriglyceridemia, and insulin resistance (Figure 1). Moreover, karyotype analysis also revealed normal 46, XX with no chromosomal abnormalities. Also, her voice was normal and not high-pitched. However, around one month before death, she developed disorientation to time and situation and had difficulty in speaking, which might be considered as the indicator of moyamoya disease or aneurysms. The patient died at the age of ~19 years old due to unknown reasons.

## Methods

No molecular genetic tests and counseling had been performed for the patient prior to referring to our center, which resulted in delay and difficulty in diagnosis and treatment of the disease. For the next step, we performed genetic variant analysis, to determine the disease and its etiology. The ethics committee of Shahid Sadoughi University of Medical Sciences confirmed this study. In addition, we obtain the written consent from the patient's parents.

### Whole Exome Sequencing

After obtaining the informed consent, genomic DNA was extracted from 100 μl of peripheral blood samples using the ReliaPrep™ kit (Blood gDNA Miniprep System, Promega) in terms of the manufacturer's instruction. Then, using the HiSeq2000 machine, whole exome sequencing (WES) was utilized in terms of the Illumina platform on DNA sample from proband (case IV-3). The coverage of the method was 100X with the sensitivity of higher than 99%.

### Bioinformatics Analysis

**Figure 2:**
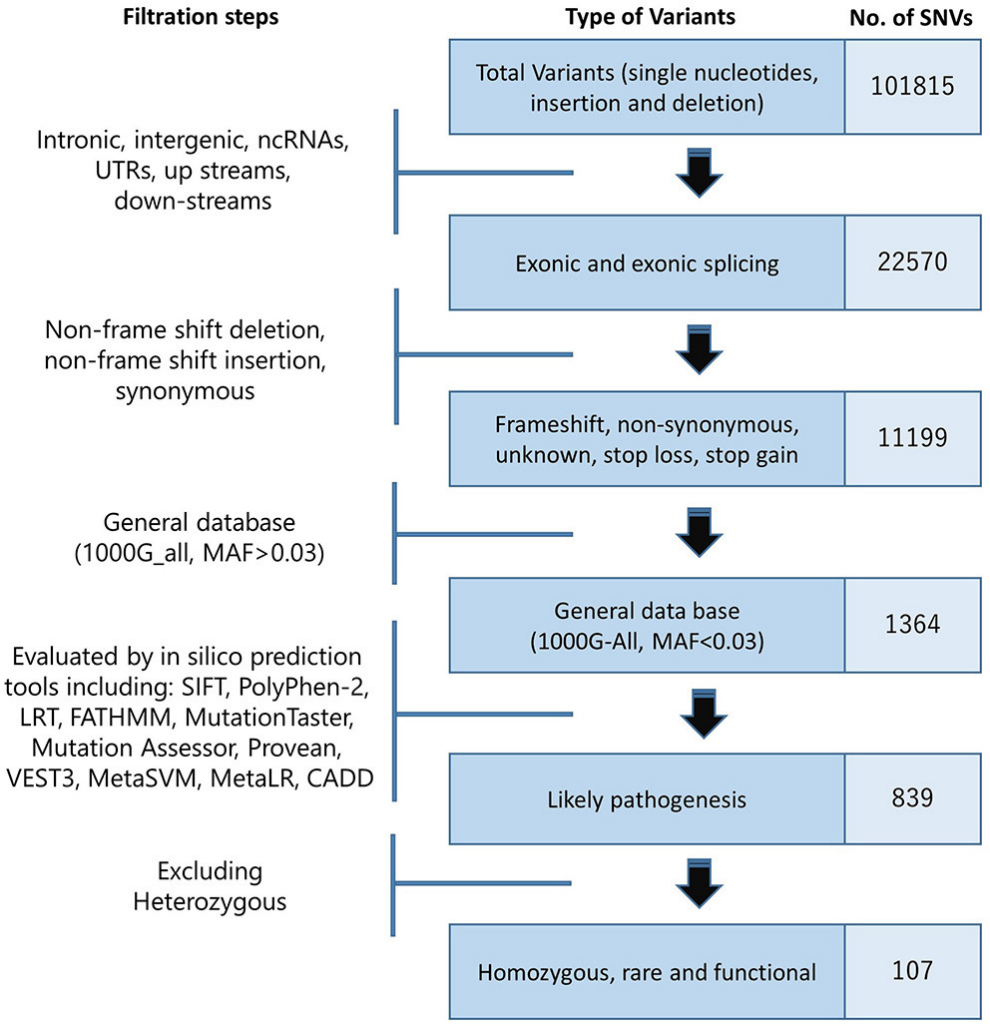
Filtrating steps to identify variant associated with the phenotype. (SNV: single-nucleotide variant).

We converted raw data (.bcl) from Hiseq2000 to fastq files by the use of bcl2fastq software (version 2.18). Next, for aligning sequences, local realignment, variants calling, and annotating; BWA (version 0.7.12) (16), GATK (version 3.5) (17), and SAM tools (18) and ANNOVAR software (19) were used, respectively. Then, we filtered 101,815 found annotated variants based on their location on the genome, and types of variation, functions, frequency, and inheritance patterns. As the phenotype was inherited in autosomal recessive, potential variant should be homozygous; therefore, we filtered the heterozygous variant. Finally, web-based phenolyzer (20) and VarElect tools (21) were employed to identify the variant associated with the phenotype and clinical features of proband from 107 homozygotes, functional, and rare variants (Figure 2).

**Figure 3:**
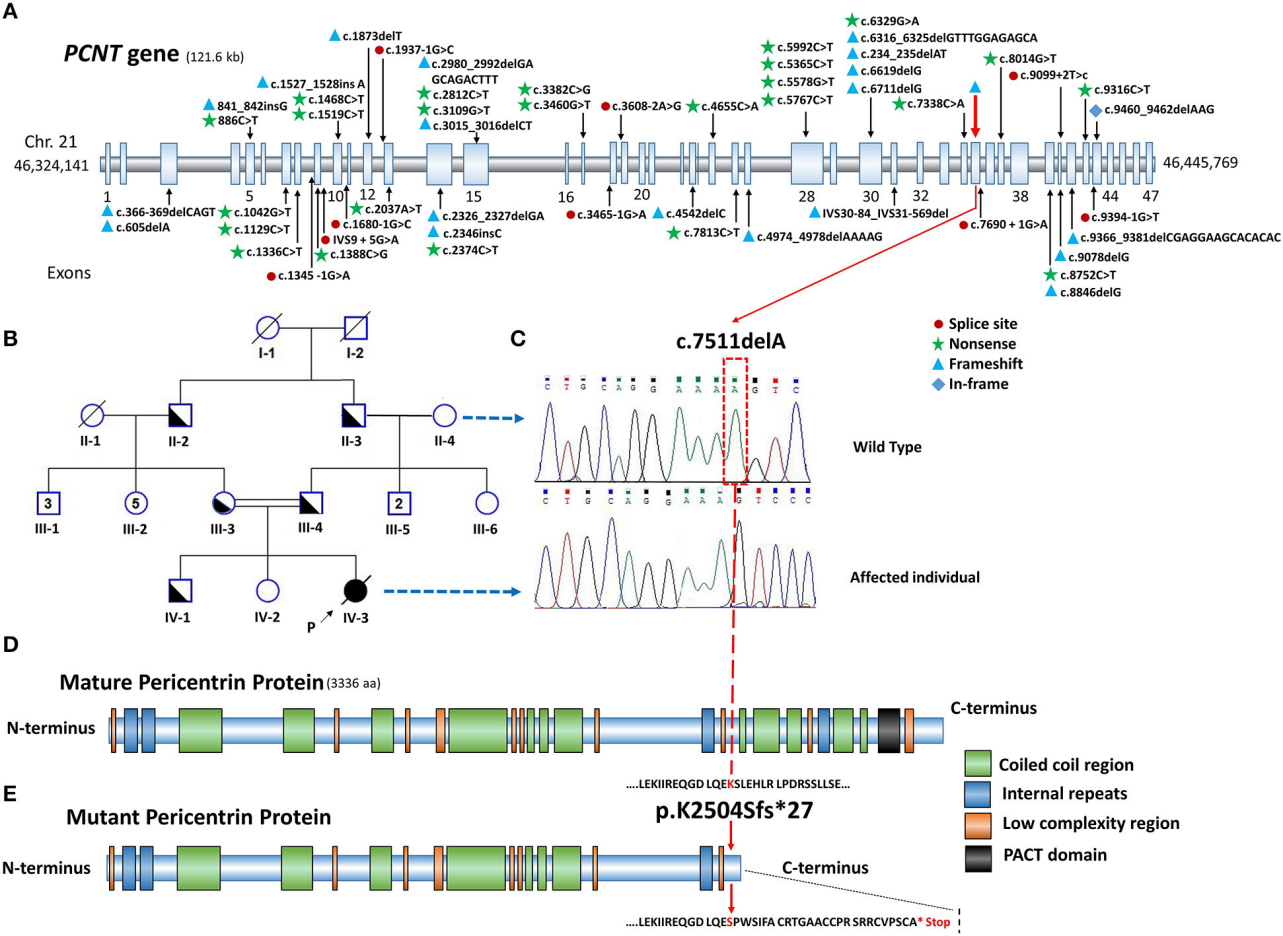
**(A)** Schematic presentation of *PCNT* gene on chromosome 21 with reported pathogenic variants associated with MOPD II. **(B)** Pedigree of patient's family, parents have consanguineous marriage. **(C)** Sanger sequencing confirmed the variant (c.7511delA) in the extended family, the patient (homozygote) and her paternal grandmother (unaffected) with corresponding chromatogram. **(D)** Schematic representation of mature Pericentrin protein with its domains and regions, data for drawing the protein domains and regions were recruited from SMART tool (Simple Modular Architecture Research Tool, http://smart.embl-heidelberg.de/, not drawn in scale). **(E)** p.K2504Sfs*27 causes premature protein with the loss of regions and domains near C terminus of protein, among which PACT domain (black domain) is a fundamental one for the appropriate function of Pericentrin protein.

After filtration analysis of all the identified variants, the results revealed a novel homozygous single base deletion (NM_006031.6:c.7511delA) in the exon 35 of the *PCNT* gene (NP_006022.3; NG_008961.2), which can creates frame-shift (p.K2504Sfs^*^27) and premature protein truncation (Figure 3E). Also, the variant has not been reported in the Exome Variant Server, Complete Genomics, Single Nucleotide Polymorphism Database (dbSNP149), 1000 Genomes, and Exome Aggregation Consortium (ExAC).

### Variant Confirmation and Segregation Analysis

In order to confirm the variant in the proband and her family, bi-directional Sanger sequencing was applied. We extracted DNA from the peripheral blood samples of the proband and for her extended family, we performed it using the aforementioned kit. Primers F 5′-CAGACTCAGCAGGCTTGTCC-3′ and R 5′-CTGCAGCTTCTCCTGGTTCT-3′ designed by Primer3.0 (http://bioinfo.ut.ee/primer3-0.4.0) were used. Then, we conducted the polymerase chain reaction (PCR) under the standard conditions. The samples were sequenced using BigDye Terminator v3.1 cycle sequencing kit and 3730 DNA Analyzer (Thermo Fisher), and the obtained results were analyzed using Finch TV and Chromas software (Figure 3C). The results confirmed the presence of the homozygote variant (c.7511delA) in the proband and heterozygote one in her parents. In addition, it was perfectly segregated within her extended family (Figure 3B).

### *In silico* Analysis

In order to predict the pathogenicity of the variant, we used different bioinformatics software and tools. The MutationTaster software (http://www.mutationtaster.org/) predicted this variant as disease-causing (Figure S1A). Furthermore, it was revealed that, PACT domain of Pericentrin protein, as a fundamental domain for its function, had been omitted based on this frame-shift variant (Figure S1B). VARSOME, which is another comprehensive online tool (https://varsome.com/), also indicated a strong pathogenicity for this variant.

## Discussion

The *PCNT* gene with 47 coding exons is located on 21q22.3 and covers ~122 kb of genomic sequence (Figure 3A). Moreover, variants in the *PCNT* gene, which codes the Pericentrin protein, can affect the cell cycle regulation and result in MOPD II. Pericentrin protein is a part of amorphous pericentriolar material (PCM), which has 3,336 amino acids that binds to calmodulin and γ-tubulin in the centrosomes and interacts with protein Kinase A and cytoplasmic dynein required for the cell-cycle progression and spindle organization (22).

Our study revealed a novel loss of function and frame-shift variant (p.K2504Sfs^*^27) in the *PCNT* gene that was perfectly segregated within the extended family. The important lost domains and regions in the premature protein due to the identified variant are depicted in Figure 3E. Therefore, it is not functional anymore, and causes clinical conditions related to the disease. It lost PACT (PCNT/AKAP9 centrosomal targeting), as a highly conserved domain at the region of a.a 3139-3216 near C-terminal, through which Pericentrin binds to the centrosome (Figure 3D) (23, 24). In addition, premature protein missed a region binding to Nek2A. Therefore, Pericentrin cannot prevent the kinase activity of Nek2, to suppress the premature centrosome splitting in interphase (25).

Since the proband had a wide variety of clinical abnormalities and due to the heterogeneity feature of the diseases associated with bone abnormalities including Seckel syndrome, we were not able to diagnose the proband disease in terms of the clinical features. Hence, we conducted WES in the proband, to identify the potential variants in genes related to the known skeletal disorders. Up to the best of our knowledge, this report has been the first genetically study on an Iranian patient suffering from MOPD II who was identified with the novel *PCNT* variant. Rauch et al. reported 29 variants scattered through the *PCNT* gene in 25 unrelated MOPD II cases (26). In another study by Willems et al., the obtained results showed 13 variants including eight MOPD II and five Seckel syndrome cases. Their study also revealed that, all the found variants would result in the loss of function (27). Also, homozygous and mixed heterozygous *PCNT* variants were identified in 4 cases from 2 unrelated Thai families suffering from MOPD II (28). The variant identified in our study is compatible with the variant identified by Piane et al. in an Italian patient, and one case that was reported by Abdel-Salam et al. (14, 29), which was frame-shift variant and resulted in the creation of a premature stop codon and a truncated protein. Up to now, based on Human Gene Mutation Database (HGMD) (www.hgmd.cf.ac.uk/), 53 different pathogenic variants causing MOPD II have been reported. These pathogenic variants are depicted in Figure 3A (6, 12–14, 26, 28–35). In addition, all variants identified in the *PCNT* gene are summarized in Table S1. Based on the reported variants, this gene has no hotspot region associated with pathogenic variants.

Microcephaly and skeletal dysplasia are recognized as major hallmarks of MOPD II that have been reported in many cases with MOPD II including the present case (29, 30, 35, 36). Generalized brachydactyly was also noted in the present case similar to the case reported by Weiss et al. (35). Clinodactyly, as another skeletal abnormality, is also prevalent among the patients with MOPD II, which was absent in the current case (29, 30). Dental abnormalities like Oligodontia, are also hallmark features of MOPD II, which were identified in the present case. Due to the Kantaputra et al. study, PCNT dysfunction is associated with the permanent dental development, since it is involved in microtubule integrity and centrosome function (28). Acanthosis nigricans is another clinical feature associated with MOPD II, which was also seen in the present patient. Several studies indicated that, this clinical feature can be associated with insulin resistance, which was also observed in many other patients suffering from MOPD II (37, 38). Although Clitoromegaly was identified in the present case, it has not been reported in the patients with MOPD II. In addition, some cases such as the cases reported by Weiss et al. (35), Ghosh et al. (30), Willems et al. (13), and Piane et al. (14) presented high-pitched voice, while the current case had normal voice. Some studies such as Ghosh et al. (30) and Hall et al. (39) reported that, the mother of the individual suffering from MOPD II may experience pregnancy with some complications such as bleeding, nausea, pre-eclampsia, premature delivery, vomiting, and fetal decelerations; however, the mother of the present case had normal pregnancy with no history of abortion. In this regard, the patient had a sudden and unknown death; however, the main cause of mortality and morbidity in these patients is ascribed to the central nervous system (CNS) of vascular anomalies (11).

The advent of Next generation sequencing (NGS) paved the way for performing the exact identification of rare and heterogeneous diseases like MOPD II, which have similar clinical conditions with other diseases. This disease is genetically homozygous, and examining NGS panels including *PCNT* gene for the individuals with prenatal and postnatal growth abnormalities and microcephaly would help pediatricians and geneticists in distinguishing this type of MOPD disease from other types. Furthermore, due to a high rate of consanguineous marriages in Iran, especially in rural areas and lack of appropriate treatment for this disease, prenatal genetic diagnosis should be taken into consideration using the NGS panel for the patients with similar phenotype.

## Conclusion

Due to recent advances in high-throughput sequencing technologies, our knowledge on the potential pathogenic variants including the variant identified in this study is increasing, which can be contributed to the improvement of genetic services such as prenatal genetic diagnosis (PND) and preimplantation genetic diagnosis (PGD). In the current study, a novel frame-shift variant in the *PCNT* gene leading to premature protein, was reported in an Iranian girl with MOPD II. Up to the best of our knowledge, this is the first report of an Iranian patient with the genetically confirmed MOPD II and a novel variant in *PCNT* gene. In addition, Sanger sequencing revealed that, the variant was perfectly segregated in the family, and some different online tools confirmed its high level of pathogenicity. Although the MOPD II disease is clinically heterogeneous and variable, it is genetically homogenous. In this regard, the result of this study extends the previous genetic findings contributing to MOPD II and emphasizes the importance of *PCNT* variants analysis, as a genetic test for performing an accurate diagnosis of MOPD II.

## Data Availability Statement

The raw data supporting the conclusions of this manuscript will be made available by the authors, without undue reservation, to any qualified researcher.

## Ethics Statement

The studies involving human participants were reviewed and approved by the ethics committee of Shahid Sadoughi University of Medical Sciences. Written informed consent to participate in this study was provided by the participants' legal guardian/next of kin. Written informed consent was obtained from the minor(s)' legal guardian/next of kin for the publication of any potentially identifiable images or data included in this article.

## Author Contributions

MD performed the experiment, analyzed the data, and wrote the manuscript. MV managed the cases, performed the experiment, and analyzed the data. HH analyzed the data. MR managed the cases and performed the clinical examination. All authors contributed to the article and approved the submitted version.

## Conflict of Interest

The authors declare that the research was conducted in the absence of any commercial or financial relationships that could be construed as a potential conflict of interest.

## References

[1] Khetarpal P Das S Panigrahi I Munshi A. Primordial dwarfism: overview of clinical and genetic aspects. Mol Genet Genomics. (2016) 291:1–15. 10.1007/s00438-015-1110-y 26323792

[2] Bongers EM Opitz JM Fryer A Sarda P Hennekam RC Hall BD. Meier-Gorlin syndrome: report of eight additional cases and review. Am J Med Genet. (2001) 102:115–24. 10.1002/ajmg.1452 11477602

[3] Boles R Teebi A Schwartz D Harper J. Further delineation of the ear, patella, short stature syndrome (Meier-Gorlin syndrome). Clin Dysmorphol. (1994) 3:207–14. 10.1097/00019605-199407000-00004 7981855

[4] Russell A. A syndrome of intra-uterine dwarfism recognizable at birth with cranio-facial dysostosis, disproportionately short arms, and other anomalies (5 examples). Proc R Soc Med. (1954) 47:1040. 13237189

[5] Majewski F Goecke T Opitz JM. Studies of microcephalic primordial dwarfism I: approach to a delineation of the Seckel syndrome. Am J Med Genet. (1982) 12:7–21. 10.1002/ajmg.1320120103 7046443

[6] Dieks J-K Baumer A Wilichowski E Rauch A Sigler M. Microcephalic osteodysplastic primordial dwarfism type II (MOPD II) with multiple vascular complications misdiagnosed as Dubowitz syndrome. Eur J Pediatr. (2014) 173:1253–6. 10.1007/s00431-014-2368-5 24973050

[7] Majewski F Goecke TO. Microcephalic osteodysplastic primordial dwarfism type II: report of three cases and review. Am J Med Genet A. (1998) 80:25–31. 10.1002/(SICI)1096-8628(19981102)80:1<25::AID-AJMG5>3.0.CO;2-0 9800908

[8] Bober MB Niiler T Duker AL Murray JE Ketterer T Harley ME. Growth in individuals with Majewski osteodysplastic primordial dwarfism type II caused by pericentrin mutations. Am J Med Genet A. (2012) 158:2719–25. 10.1002/ajmg.a.35447 22821869

[9] Sigaudy S Toutain A Moncla A Fredouille C Bourliere B Ayme S. Microcephalic osteodysplastic primordial dwarfism Taybi-Linder type: report of four cases and review of the literature. Am J Med Genet A. (1998) 80:16–24. 10.1002/(SICI)1096-8628(19981102)80:1<16::AID-AJMG4>3.0.CO;2-5 9800907

[10] Engel M Castrillon-Oberndorfer G Hoffmann J Egermann M Freudlsperger C Thiele OC. Cranial vault remodeling in microcephalic osteodysplastic primordial dwarfism type II and craniosynostosis. J Craniofac Surg. (2012) 23:1407–9. 10.1097/SCS.0b013e31825e4b18 22948629

[11] Bober MB Jackson AP. Microcephalic osteodysplastic primordial dwarfism, type II: a clinical review. Curr Osteoporos Rep. (2017) 15:61–9. 10.1007/s11914-017-0348-1 28409412 PMC5561166

[12] Unal S Alanay Y Cetin M Boduroglu K Utine E Cormier-Daire V. Striking hematological abnormalities in patients with microcephalic osteodysplastic primordial dwarfism type II (MOPD II): a potential role of pericentrin in hematopoiesis. Pediatr Blood Cancer. (2014) 61:302–5. 10.1002/pbc.24783 24106199

[13] Willems M Genevieve D Borck G Baumann C Baujat G Bieth E. Molecular analysis of pericentrin gene (PCNT) in a series of 24 Seckel/microcephalic osteodysplastic primordial dwarfism type II (MOPD II) families. J Med Genet. (2010) 47:797–802. 10.1136/jmg.2009.067298 19643772

[14] Piane M Della Monica M Piatelli G Lulli P Lonardo F Chessa L. Majewski osteodysplastic primordial dwarfism type II (MOPD II) syndrome previously diagnosed as Seckel syndrome: report of a novel mutation of the PCNT gene. Am J Med Genet A. (2009) 149:2452–6. 10.1002/ajmg.a.33035 19839044

[15] Flory MR Moser MJ Monnat RJ Davis TN. Identification of a human centrosomal calmodulin-binding protein that shares homology with pericentrin. PNAS. (2000) 97:5919–23. 10.1073/pnas.97.11.5919 10823944 PMC18534

[16] Li H Durbin R. Fast and accurate short read alignment with burrows-Wheeler transform. Bioinformatics. (2009) 25:1754–60. 10.1093/bioinformatics/btp324 19451168 PMC2705234

[17] McKenna A Hanna M Banks E Sivachenko A Cibulskis K Kernytsky A. The genome analysis toolkit: a MapReduce framework for analyzing next-generation DNA sequencing data. Genome Res. (2010) 20:1297–303. 10.1101/gr.107524.110 20644199 PMC2928508

[18] Li H Handsaker B Wysoker A Fennell T Ruan J Homer N. The sequence alignment/map format and SAMtools. Bioinformatics. (2009) 25:2078–9. 10.1093/bioinformatics/btp352 19505943 PMC2723002

[19] Wang K Li M Hakonarson H. ANNOVAR: functional annotation of genetic variants from high-throughput sequencing data. Nucleic Acids Res. (2010) 38:e164–e. 10.1093/nar/gkq603 20601685 PMC2938201

[20] Yang H Robinson PN Wang K. Phenolyzer: phenotype-based prioritization of candidate genes for human diseases. Nat Methods. (2015) 12:841. 10.1038/nmeth.3484 26192085 PMC4718403

[21] Stelzer G Plaschkes I Oz-Levi D Alkelai A Olender T Zimmerman S. VarElect: the phenotype-based variation prioritizer of the GeneCards Suite. BMC Genomics. (2016) 17:444. 10.1186/s12864-016-2722-2 27357693 PMC4928145

[22] Zimmerman WC Sillibourne J Rosa J Doxsey SJ. Mitosis-specific anchoring of γ tubulin complexes by pericentrin controls spindle organization and mitotic entry. Mol Biol Cell. (2004) 15:3642–57. 10.1091/mbc.e03-11-0796 15146056 PMC491825

[23] Gillingham AK Munro S. The PACT domain, a conserved centrosomal targeting motif in the coiled-coil proteins AKAP450 and pericentrin. EMBO Rep. (2000) 1:524–9. 10.1093/embo-reports/kvd105 11263498 PMC1083777

[24] Watanabe K Takao D Ito KK Takahashi M Kitagawa D. The Cep57-pericentrin module organizes PCM expansion and centriole engagement. Nat Commun. (2019) 10:931. 10.1038/s41467-019-08862-2 30804344 PMC6389942

[25] Matsuo K Nishimura T Hayakawa A Ono Y Takahashi M. Involvement of a centrosomal protein kendrin in the maintenance of centrosome cohesion by modulating Nek2A kinase activity. Biochem Biophys Res Commun. (2010) 398:217–23. 10.1016/j.bbrc.2010.06.063 20599736

[26] Rauch A Thiel CT Schindler D Wick U Crow YJ Ekici AB. Mutations in the pericentrin (PCNT) gene cause primordial dwarfism. Science. (2008) 319:816–9. 10.1126/science.1151174 18174396

[27] Willems M Genevieve D Borck G Baujat G Gerard M Heron D. editors. Pericentrin molecular analysis in 22 Seckel/MOPDII patients. In: 58th Annual Meeting, American Society of Human Genetics. Philadelphia, PA (2008).

[28] Kantaputra P Tanpaiboon P Porntaveetus T Ohazama A Sharpe P Rauch A. The smallest teeth in the world are caused by mutations in the PCNT gene. Am J Med Genet A. (2011) 155:1398–403. 10.1002/ajmg.a.33984 21567919

[29] Abdel-Salam GM Sayed IS Afifi HH Abdel-Ghafar SF Abouzaid MR Ismail SI. Microcephalic osteodysplastic primordial dwarfism type II: additional nine patients with implications on phenotype and genotype correlation. Am J Med Genet A. (2020) 182:1407–20. 10.1002/ajmg.a.61585 32267100

[30] Ghosh S Garg M Gupta S Choudhary M Chandra M. Microcephalic osteodyplastic primordial dwarfism type II: case report with unique oral findings and a new mutation in the pericentrin gene. Oral Surg Oral Med Oral Pathol Oral Radiol Endod. (2019) 129–e204–11. 10.1016/j.oooo.2019.08.019 31606423

[31] Pachajoa H Ruiz-Botero F Isaza C. A new mutation of the PCNT gene in a Colombian patient with microcephalic osteodysplastic primordial dwarfism type II: a case report. J Med Case Rep. (2014) 8:191. 10.1186/1752-1947-8-191 24928221 PMC4086705

[32] Faienza MF Acquafredda A D'Aniello M Soldano L Marzano F Ventura A. Effect of recombinant insulin-like growth factor-1 treatment on short-term linear growth in a child with Majewski osteodysplastic primordial dwarfism type II and hepatic insufficiency. J Pediatr Endocrinol Metab. (2013) 26:771–4. 10.1515/jpem-2012-0397 23612698

[33] Kantaputra PN. Apparently new osteodysplastic and primordial short stature with severe microdontia, opalescent teeth, and rootless molars in two siblings. Am J Med Genet. (2002) 111:420–8. 10.1002/ajmg.10589 12210304

[34] Shaheen R Maddirevula S Ewida N Alsahli S Abdel-Salam GM Zaki MS. Genomic and phenotypic delineation of congenital microcephaly. Genet Med. (2019) 21:545–52. 10.1038/s41436-018-0140-3 30214071 PMC6986385

[35] Weiss K Ekhilevitch N Cohen L Bratman-Morag S Bello R Martinez AF. Identification of a novel PCNT founder pathogenic variant in the Israeli Druze population. Eur J Med Genet. (2020) 63:103643. 10.1016/j.ejmg.2019.03.007 30922925

[36] Vakili R Hashemian S. Primordial dwarfism: a case series from north east of Iran and literature review. J Pediatr Rev. (2019) 7:113–20. 10.32598/jpr.7.2.113

[37] Hermanns-Lê T Scheen A Piérard GE. Acanthosis nigricans associated with insulin resistance. Am J Clin Dermatol. (2004) 5:199–203. 10.2165/00128071-200405030-00008 15186199

[38] Huang-Doran I Bicknell LS Finucane FM Rocha N Porter KM Tung YL. Genetic defects in human pericentrin are associated with severe insulin resistance and diabetes. Diabetes. (2011) 60:925–35. 10.2337/db10-1334 21270239 PMC3046854

[39] Hall JG Flora C Scott Jr CI Pauli RM Tanaka KI. Majewski osteodysplastic primordial dwarfism type II (MOPD II): natural history and clinical findings. Am J Med Genet A. (2004) 130:55–72. 10.1002/ajmg.a.30203 15368497

