# Supplementary figures and images for: A Novel PCNT Frame Shift Variant (c.7511delA) Causing Osteodysplastic Primordial Dwarfism of Majewski Type 2 (MOPD II)
Source: Front Pediatr. 2020 Jun 25;8:340. doi: 10.3389/fped.2020.00340 (PMC7330014; doi:10.3389/fped.2020.00340)

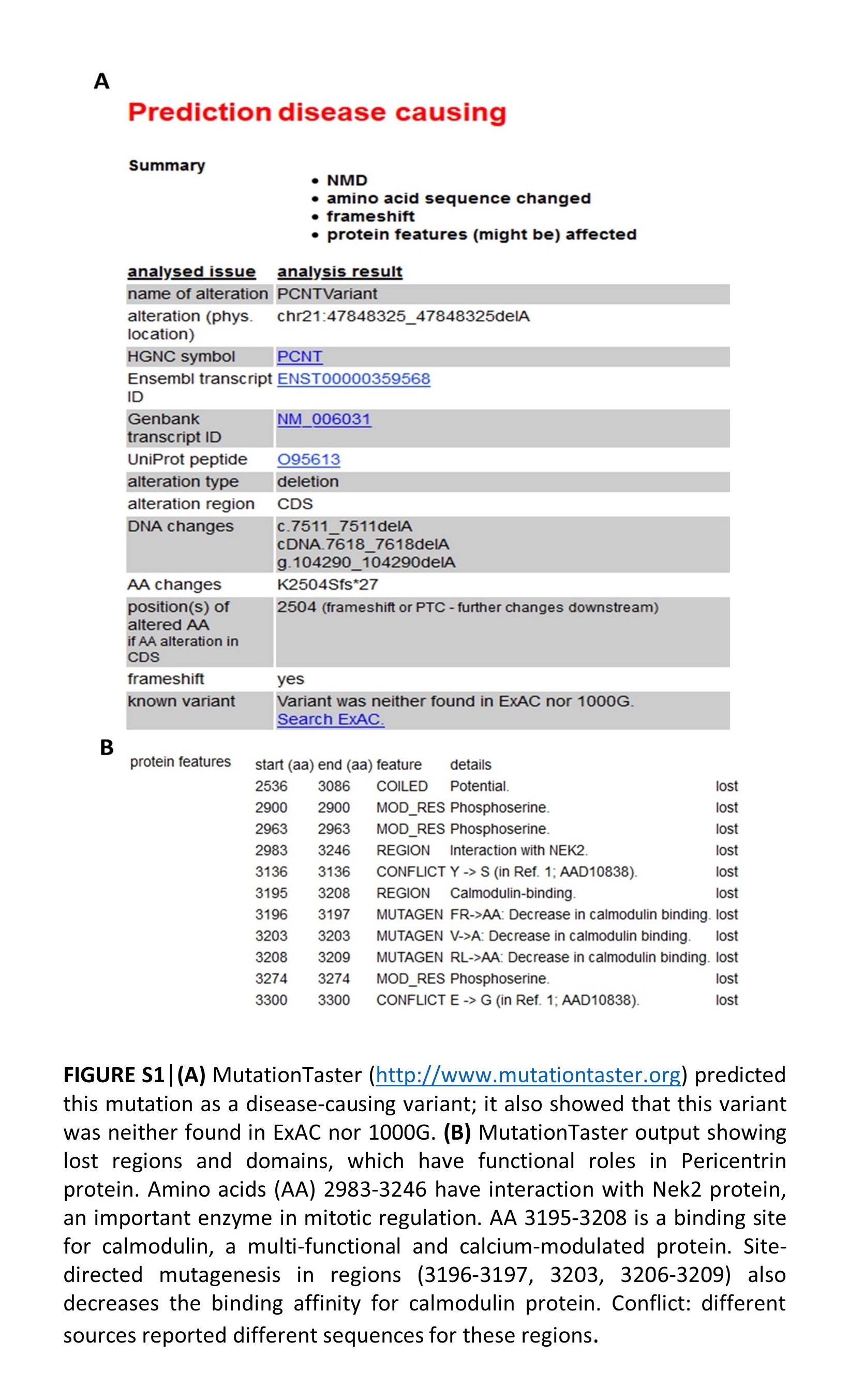

Supplement: Supplementary file 2 [file Image_1.jpg]
